# A database for the monitoring of thermal anomalies over the Amazon forest and adjacent intertropical oceans

**DOI:** 10.1038/sdata.2015.24

**Published:** 2015-05-26

**Authors:** Juan C. Jiménez-Muñoz, Cristian Mattar, José A. Sobrino, Yadvinder Malhi

**Affiliations:** 1 Global Change Unit/Image Processing Laboratory, University of Valencia, Valencia 46980, Spain; 2 Laboratory for Analysis of the Biosphere, University of Chile, Santiago 11315, Chile; 3 Environmental Change Institute, University of Oxford, Oxford OX1 3QY, UK

**Keywords:** Forest ecology, Climate-change impacts, Tropical ecology

## Abstract

Advances in information technologies and accessibility to climate and satellite data in recent years have favored the development of web-based tools with user-friendly interfaces in order to facilitate the dissemination of geo/biophysical products. These products are useful for the analysis of the impact of global warming over different biomes. In particular, the study of the Amazon forest responses to drought have recently received attention by the scientific community due to the occurrence of two extreme droughts and sustained warming over the last decade. *Thermal Amazoni@* is a web-based platform for the visualization and download of surface thermal anomalies products over the Amazon forest and adjacent intertropical oceans using Google Earth as a baseline graphical interface (http://ipl.uv.es/thamazon/web). This platform is currently operational at the servers of the University of Valencia (Spain), and it includes both satellite (MODIS) and climatic (ERA-Interim) datasets. *Thermal Amazoni@* is composed of the viewer system and the web and ftp sites with ancillary information and access to product download.

## Background & Summary

The resilience of tropical forests to more frequent drought events and sustained warming is a major concern because of its impact on the absorption of atmospheric carbon dioxide. In particular, the Amazon forests include more than 50% of the world’s tropical forests and stores more than 100 billion tonnes of carbon^1^, so they are a key component of the global carbon cycle. The forest capability of absorbing CO_2_ can be reduced under different environmental conditions, as for example anomalous warming or heat stress. Some studies found a correlation between the decrease in tropical forest productivity and the increase in temperature, thus reducing the capability of carbon uptake and favoring the accumulation of atmospheric CO_2_ (ref. 2), while other studies suggested that tropical forests are near a high temperature threshold above which CO_2_ uptake drops sharply^3^.

Anomalous high temperatures can be reached under extreme dry conditions. The occurrence of drought events is one major aspect of Amazonian climate change^4^. Attention was paid to Amazonia because of two major droughts in 2005 (ref. 5) and 2010 (ref. 6), which were considered amongst the most severe in a century in such a short time of period. These drought events have been associated with increased tree mortality and losses of biomass, and also with temporary shutdown of the Amazon carbon sink^7–10^. Moreover, a conversion of Amazonian tropical forest to savanna due to a sustained warming and more frequent droughts events has also been predicted^11^.

Some studies analyzed the link between droughts and Sea Surface Temperature (SST) anomalies over the central Pacific (El Niño events) and the Tropical North Atlantic (TNA). El Niño events play an important role in the wet-season rainfall, since it suppresses convections in northern and eastern Amazonia, whereas the dry-season rainfall is influenced by the warming in the TNA SST, since it strengthens the Hadley Cell circulation and trade winds are weaken^4,5^. The influence of SST anomalies over the warming in Amazon has been found to be dependent on the Amazonian region and season^12^.

The study of the Amazon forest responses to drought has been extensively reported in the literature in the last years through the analysis of anomalies in vegetation indices obtained from remote sensing data. However, recent analysis of Land Surface Temperature (LST) anomalies over the Amazon forest is very scarce^8,12^. Since anomalous high temperatures can be more important than precipitation deficits in causing the losses of biomass^13^, the monitoring of thermal anomalies can provide valuable information about the interactions between the Amazonian climate and other factors which may induce a widespread degradation of the Amazonian forests (e.g., fires, deforestation).

Thermal Amazoni@ was developed to disseminate maps of thermal anomalies over the Amazon forest and adjacent intertropical oceans in a user-friendly manner, even for the non-scientific public, and also to facilitate the scientific analysis of thermal anomalies and trends in vegetation temperature, as well as its relationship with SST anomalies over particular intertropical regions. For this purpose, the system includes the digital maps to be visualized, but also the raw data from which the maps were generated in order to allow the scientific users their own processing or analysis.

## Methods

### Satellite and reanalysis data

Satellite imagery includes data acquired with the Moderate Resolution Imaging Spectroradiometer (MODIS) on board de NASA’s Terra platform. In particular, the monthly LST product MOD11C3 at 0.05° latitude/longitude Climate Modeling Grid (CMG) version 5 was used^14^. This product is available from year 2000 to present, and it includes both daytime and nighttime monthly averages of LST. Cloud contamination affects the computation of the monthly LST value because of failures of the MODIS Cloud Mask^15^. Therefore, Thermal Amazoni@ also includes a new reprocessing of the daily MODIS LST product (MOD11C1) to generate a new monthly LST after removal of outliers (±3σ criterion). This new product is denoted as MODrep, and the mean LST is only computed if at least 50% of days within a month provide valid data. The Combined Terra/Aqua yearly Land Cover product (MCD12C1 version 5.1) available from 2001 to 2012 (ref. 16) is also included to identify pixels classified as Evergreen Broadleaf Forest (EBF).

The reanalysis data included monthly means of skin temperature extracted from the ERA-Interim project developed by the European Centre for Medium-Range Weather Forecasts (ECMWF) at 0.75°×0.75° latitude longitude global spatial resolution^17^. The ERA-interim skin temperature product is the temperature used in the derivation of the heat budget between the atmosphere and the surface, in which a zero heat flux condition is set at the bottom as a boundary condition^18^. Therefore, satellite data is not assimilated in the estimation of the skin temperature, since this product is directly derived from the energy budget of the simulated grid.

### Computation of thermal anomalies and trends

LST values (or SST values) were converted to absolute (abs) and standardized (std) anomalies (a), expressed as

(1)aLSTabs=LST−LSTmean

(2)aLSTstd=LST−LSTmeanσ=aLSTabsσ

where LST_mean_ is the mean temperature over a reference period (climatological mean), and σ is the standard deviation of the climatological mean. Standardized anomalies are considered analogous to the z-score, so they can be easily interpreted in terms of probability of a certain value being anomalous. Thermal anomalies are calculated at different time levels: monthly, seasonal and yearly. In the case of seasonal anomalies, the four seasons January-February-March (JFM), April-May-June (AMJ), July-August-September (JAS), and October-November-December (OND) are considered.

Trends in temperature anomalies are also computed. For this purpose, a Mann-Kendall analysis^19^ is used to identify the significance of the trend, whereas the Sen’s method^20^ is used to estimate the slope (warming/cooling rate). These methods are nonparametric and make no assumptions on distribution of data. As a general rule, a confidence level of 95% (α=0.05) is considered statistically significant.

### System architecture

Thermal Amazoni@ system is currently installed at the servers of the Image Processing Laboratory (IPL) of the University of Valencia (Spain), but it is highly portable and can be easily installed in any other server. The system is composed of three modules: (i) the viewer, (ii) the web site, and (iii) the ftp site.

The viewer module is available at http://ipl.uv.es/thamazon, and it allows the visualization of the thermal anomalies maps in a Google Earth interface. The development of this module is based in Open Source codes. All the algorithms for viewing and browsing the imagery included in the system were developed entirely in Javascript (http://www.ecmascript.org) and Hyper Text Markup Language version 5 (HTML5, http://www.w3.org). Underlying libraries for map drawing are OpenLayers based (http://www.openlayers.org), whereas rich interface components (trees, forms, buttons, menus, etc.) were built on top of Sencha ExtJS library (http://www.sencha.com/products/extjs/). The functionality of OpenLayers was combined with the user interface of the Sencha ExtJS library using GeoExt2 library (http://geoext.github.io/geoext2). The imagery database definition was built using an *ad-hoc* Extensible Markup Language (XML, http://www.w3.org/xml) format, bringing a flexible data model to allow easy programmatic generation. In order to improve system portability and simplicity neither Structured Query Language (SQL) nor NoSQL database were used.

The web site is available at http://ipl.uv.es/thamazon/web, and it includes different information about the Thermal Amazoni@ project, news and updates, downloads, etc. The File Transfer Protocol (FTP) site allows the download of the different maps and also products in raw data. Users can access the FTP site using any ftp client with the following information (no password is required): server: *ipl.uv.es*, user: *ftpthamazon*. It is also possible to access the FTP site using a web browser at the following link: ftp://ftpthamazon@ipl.uv.es.

### Known problems in the access to the viewer

Thermal Amazoni@ Viewer is optimized to be accessed through the most modern version of different browers: Google Chrome 40.0, Mozilla Firefox 35.0, Internet Explorer 11, Safari 7.0 (or higher versions). Except for Internet Explorer, all these navigators are multiplatform and can be acquired for free. The viewer has been successfully tested under different Operating Systems (OS) such as Windows and Mac OS X, as well as different Linux based OS, such as Ubuntu 14.0, CentOS 7.0, Kali Linux 1.1.0 (and associated browsers such as Chromium 41.0 or Iceweasel 31.5.0). Access to the Viewer using older versions of the navigators may fail, and the following message errors may arise: ‘h.getMessage is not a function’ or ‘Object doesn’t support this property or method’. Since the Viewer was developed to fully exploit the features of the Open Source codes presented in the previous section, it cannot be accessed using old versions of internet navigators.

Another known problem when accessing the viewer is related to the time required to load all the imagery to be visualized. The current version of the viewer first loads all the imagery before they can be visualized. Depending on the technical characteristics of the computer and the speed of the internet connection, this loading time may be high for some browsers, so that some error message may arise. In despite of this error message, the Viewer is actually working, so It does not prevent the user to access the viewer.

It should be noted that no problems have been found for the access to the web site (http://ipl.uv.es/thamazon/web) and to the ftp site, independently of the navigator version used. Moreover, the data can be also accessed through Figshare (Data Citation 1, Data Citation 2 and Data Citation 3).

## Data Records

Data records included in the Thermal Amazoni@ *Viewer* module are absolute temperature anomalies, warming index, trends (slope) in temperature anomalies, and land cover (Table 1). Data records are provided for the Amazon basin (45W-80W, 10N-20S; pixels classified as EBF), and also for the tropical sea regions of the Pacific and Atlantic. The warming index is included to facilitate the interpretation of the anomalies maps, and it is defined from probability ranges extracted from the values of standardized anomalies (Table 2). The levels of warming were defined using the criteria considered in other studies^21^.

The different products can be visualized through the viewer system (http://ipl.uv.es/thamazon) and downloaded via the ftp server (see http://ipl.uv.es/thamazon/web, ‘FTP site’ section). MODIS data sets (Data Citation 1 and Data Citation 2) and ERA-Interim data sets (Data Citation 3) are also available to the public through the figshare repository. Raw products are provided in GeoTIFF format, with a ‘Geographic Lat/Lon’ projection and datum WGS-84. The size of the images (samples×files) are 701×601 and 48×42 for MODIS and ERA-interim surface temperature, respectively. In the case of SST anomalies from ERA-Interim, the size is 227×65. Data values are provided in floating point format. Figures of maps and legends are also included in the ftp server in PNG format. Moreover, graphs of time series of monthly and seasonal thermal anomalies for each pixel are included in the ftp server in PNG format. Graphs filenames include the sample and file which can be easily converted to (lat,lon) coordinates using an excel file also included in the ftp.

LST products are monthly updated (when available), and thermal anomalies maps and graphs are consequently updated and upload to the system. Although currently Thermal Amazoni@ is focused on LST products, other products can be easily incorporated to the system. It is also expected to include *in situ* data for calibration and validation of LST products.

## Technical Validation

**Figure 1:**
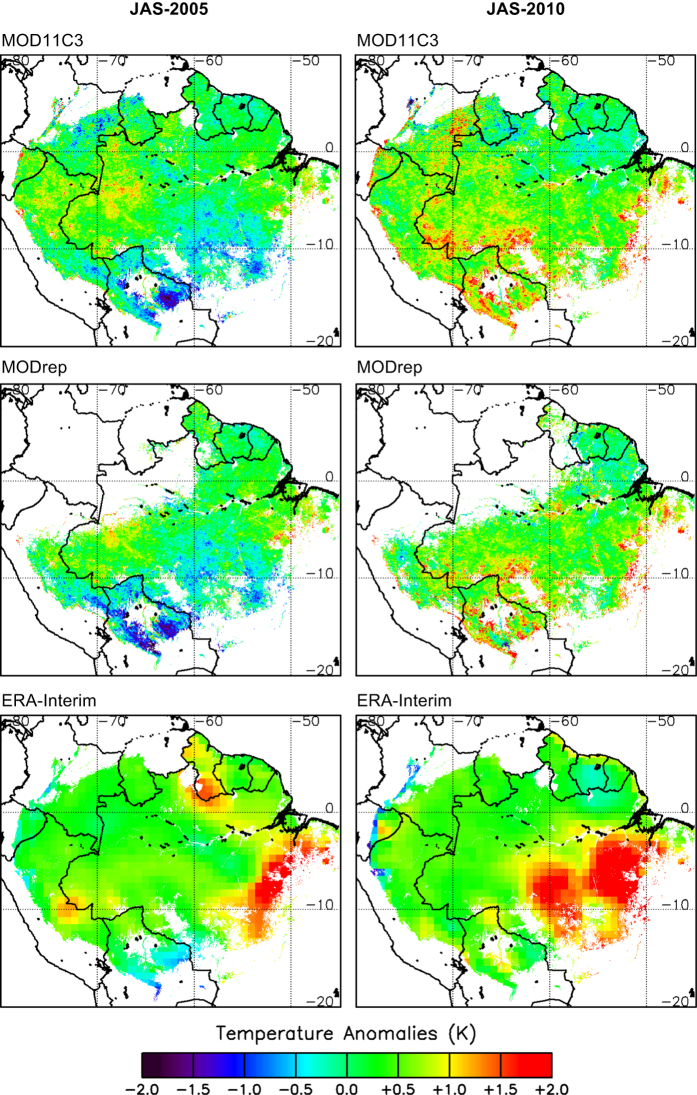
Thermal anomalies over the Amazon forest for the July-August-September (JAS) season in years 2005 and 2010. Maps of thermal anomalies were obtained from MODIS products (standard product MOD11C3 and the reprocessed product MODrep) and ERA-Interim products.

**Figure 2:**
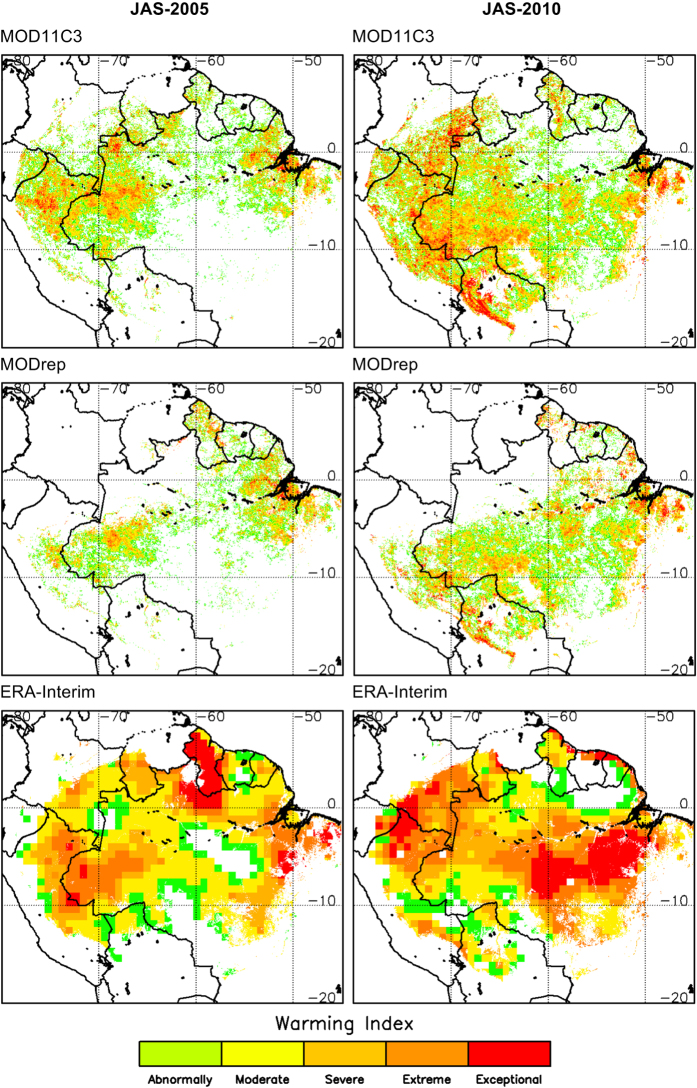
Warming index over the Amazon forest for the July-August-September (JAS) season in years 2005 and 2010. Maps of warming index were obtained from MODIS products (standard product MOD11C3 and the reprocessed product MODrep) and ERA-Interim products.

Thermal anomalies maps included in Thermal Amazoni@ obtained from MODIS and ERA-Interim data are presented in Fig. 1 for some illustrative examples. Figure 2 includes the maps of warming index for the examples presented in Fig. 1. These figures illustrate the anomalous warming for the two severe droughts in 2005 and 2010, especially enhanced in the maps of thermal anomalies obtained from ERA-Interim. Another aspect observed in these figures is the reduction on the number of valid pixels in the MODrep product, due to the outliers removal in the reprocessing and the condition of enough number of days with valid data within a month to compute the monthly mean. This reduction in the spatial coverage of the MODrep product is mainly attributed to quasi permanent cloudy conditions over particular regions of Amazonia, and it is even more dramatic in the JFM season.

**Figure 3:**
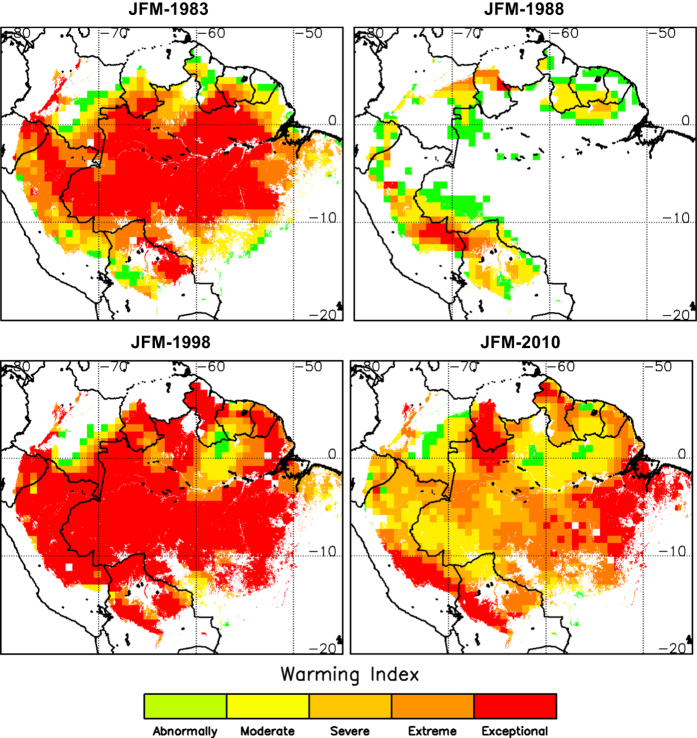
Warming index over the Amazon forest for the January-February-March (JFM) season. Maps are provided for years 1983, 1988, 1998 and 2010, when moderate or severe El Niño events took place. Maps of warming index were obtained from ERA-Interim products.

**Figure 4:**
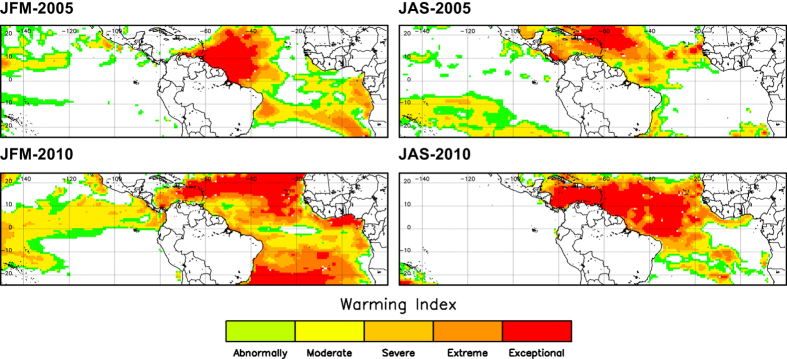
Warming index over the tropical Pacific and Atlantic sea regions for the JFM and JAS seasons. Maps are provided for years 2005 and 2010, when the two severe droughts over Amazonia took place. Maps of warming index were obtained from ERA-Interim products.

Figure 3 shows the warming index for the JFM season and years when moderate or strong El Niño events took place, such as 1983, 1988, 1998, and 2010. It is clearly observed the anomalous warming over Amazonia induced by El Niño episodes. Figure 4 includes SST anomalies (a product also included in Thermal Amazoni@) obtained from ERA-Interim in 2005 and 2010 for the seasons JFM and JAS. Anomalous warming in the tropical North Atlantic (and also in the tropical South Atlantic in the case of JFM-2010) is observed. This particular feature was linked to the severe drought of 2005 and 2010 over Amazonia, as a different case of the El Niño events when the tropical Pacific is abnormally warmed.

**Figure 5:**
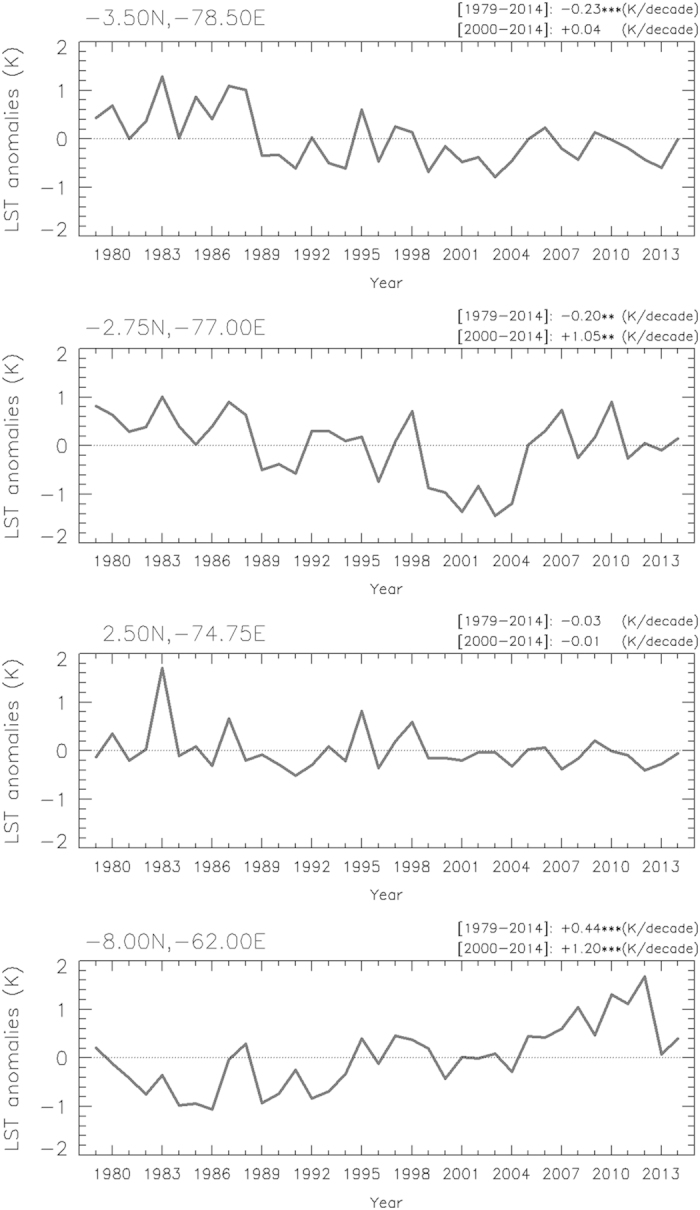
Time series of thermal anomalies since 1979 for the JAS season and over particular pixels in the Amazon forests. Values were obtained from ERA-Interim anomalies, and the slope of the trend (in °C/decade) is also provided for two periods, 1979–2014 and 2000–2014. The asterisk refers to the level of significance (*P<0.10, **P<0.05, ***P<0.01).

Thermal Amazoni@ also includes graphs of time series of thermal anomalies over particular pixels (identified by geographical coordinates of latitude and longitude). These graphs provide the slope of the trend and its significance level for two periods, 1979–2014 and 2000–2014. Figure 5 includes time series of thermal anomalies for the JAS season over four arbitrarily selected pixels.

## Usage Notes

Thermal anomalies provide valuable information in order to study the impact of drought events on the Amazon forests. They can be used with other ancillary data (e.g., measurements of biomass, measurements of CO_2_ fluxes, etc.) to assess the impact of anomalous warming on the forests physiological behaviour.

Raw data can be used to reprocess the thermal anomalies (e.g., using other periods for the climatological mean), elaborate new warming indices, etc. The different maps and graphs already included in the system can be used for trend analysis. Figures are included with geographical boundaries to be used in scientific reports or papers, but also they are included with a transparent background and without geographical boundaries or other gridlines, so they can be used in other visualization systems. Moreover, the visualization tool allows the overlapping of products with different degrees of transparency, a useful tool for the intercomparison of the different products. Maps of thermal anomalies over Amazonia and thermal anomalies over the different sea regions can be also overlapped, thus allowing a complete analysis of the link between SST anomalies and warming over the Amazon forest.

## Additional information

**How to cite this article:** Jiménez-Muñoz, J. C., Mattar, C., Sobrino, J. A. & Malhi, Y. A database for the monitoring of thermal anomalies over the Amazon forest and adjacent intertropical oceans. *Sci. Data* 2:150024 doi: 10.1038/sdata.2015.24 (2015).

## Supplementary Material



## Figures and Tables

**Table 1:** Products included in the Thermal Amazoni@ system.

**Layer**	**Source**	**Availability**	**Resolution**	**Parameter**	**Units**
LSTday_MOD11C3v5	MODIS	2000-present	0.05	Anomalies	°C (or K)
				Warming Index	Unitless
				Trend (Slope)	°C/dec (or K/dec)
LSTday_MODrep	MODIS	2000-present	0.05	Anomalies	°C (or K)
				Warming Index	Unitless
				Trend (Slope)	°C/dec (or K/dec)
SKT_ERAint	ERA-int	1979-present	0.75	Anomalies	°C (or K)
				Warming Index	Unitless
				Trend (Slope)	°C/dec (or K/dec)
SST_ERAint	ERA-int	1979-present	0.75	Anomalies	°C (or K)
				Warming Index	Unitless
All products are provided at monthly and seasonal levels. LST: Land Surface Temperature; day: daytime acquisition; MOD: MODIS; SKT: Skin temperature; ERAint: ECMWF ERA Interim reanalysis.					

**Table 2:** Definition of levels of warming included in the Warming Index product from the values of standardized anomalies.

**Standardized anomaly**	**Probability (%)**	**Level of warming**
+0.5 to +0.8	39.1 to 57.6	Abnormally
+0.8 to +1.3	57.6 to 80.4	Moderate
+1.3 to +1.6	80.4 to 89	Severe
+1.6 to +2	89 to 95.4	Extreme
+2 or high	>95.4	Exceptional

## References

[d1] Figshare Jiménez-Muñoz J. C. Mattar C. Sobrino J. A. Malhi Y. 2015 http://dx.doi.org/10.6084/m9.figshare.1304528 10.1038/sdata.2015.24 PMC4443878 26029379

[d2] Figshare Jiménez-Muñoz J. C. Mattar C. Sobrino J. A. Malhi Y. 2015 http://dx.doi.org/10.6084/m9.figshare.1304526 10.1038/sdata.2015.24 PMC4443878 26029379

[d3] Figshare Jiménez-Muñoz J. C. Mattar C. Sobrino J. A. Malhi Y. 2015 http://dx.doi.org/10.6084/m9.figshare.1304857 10.1038/sdata.2015.24 PMC4443878 26029379

